# Next-generation therapies for osteoarthritis: the evolving role of cell therapy products

**DOI:** 10.1038/s12276-026-01726-y

**Published:** 2026-06-10

**Authors:** Valtteri Peitso, Ron Ellis, Karman Ng, Simone Ponta, Rana Smaida, Nadia Benkirane-Jessel, Nancy P. Duarte-Delgado, Tobias Winkler, Goncalo Barreto, Jean-Yves Reginster, Ali Mobasheri

**Affiliations:** 1 Research Unit of Health Sciences and Technology, Faculty of Medicine, University of Oulu, Oulu, Finland; 2 Independent Researcher, Tampa, FL USA; 3 Tissue Engineering + Biofabrication Laboratory, Department of Health Sciences and Technology, ETH Zürich, Zürich, Switzerland; 4 Lamina Therapeutics, Strasbourg, France; 5 Institut National de la Santé et de la Recherche Médicale (INSERM), UMR 1260, Regenerative NanoMedicine (RNM), Strasbourg, France; 6 Université de Strasbourg, Strasbourg, France; 7 Center for Musculoskeletal Surgery, Corporate Member of Freie Universität Berlin and Humboldt-Universität zu Berlin, Charité — Universitätsmedizin Berlin, Berlin, Germany; 8 Julius Wolff Institute, Berlin Institute of Health, Charité — Universitätsmedizin Berlin, Berlin, Germany; 9 Berlin Institute of Health Center for Regenerative Therapies, Corporate Member of Freie Universität Berlin, Humboldt Universität zu Berlin and Berlin Institute of Health, Charité — Universitätsmedizin Berlin, Berlin, Germany; 10 Department of Orthopedics and Trauma Surgery, Medical University of Vienna, Vienna, Austria; 11 Clinicum, Faculty of Medicine, University of Helsinki and Helsinki University Hospital, Helsinki, Finland; 12 Protein Research Chair, Department of Biochemistry, College of Science, King Saud University, Riyadh, Kingdom of Saudi Arabia; 13 Department of Personalized Medicine, State Research Institute Centre for Innovative Medicine, Vilnius, Lithuania; 14 Department of Joint Surgery, First Affiliated Hospital of Sun Yat-sen University, Guangzhou, China; 15 Faculty of Medicine, Université de Liège, Liège, Belgium; 16 Department for Health Sciences, Medicine and Research, Center for Regenerative Medicine, Faculty of Health and Medicine, University for Continuing Education Krems, Krems, Austria; 17 Department of Orthopedic Surgery, Maastricht University Medical Center, Maastricht University, Maastricht, Netherlands

**Keywords:** Stem-cell research, Translational research, Regenerative medicine, Stem cells, Osteoarthritis

## Abstract

Osteoarthritis (OA) remains a major cause of disability worldwide; however, current non-surgical treatments offer transient symptom relief without altering disease course. This leaves a therapeutic gap for patients with early-to-moderate disease who are not candidates for surgery but continue to experience pain and functional limitation. Intra-articular interventions such as non-steroidal anti-inflammatory drugs, hyaluronic acid, and platelet-rich plasma may ease symptoms, but do not modify disease progression. By contrast, cell therapy products hold promise as regenerative approaches that may both alleviate pain and influence disease trajectory. Cell therapy products for knee OA exert multimodal effects through paracrine and immunomodulatory mechanisms, including modulation of synovial inflammation, attenuation of senescence-associated pathways, and support of extracellular matrix production. Despite encouraging preclinical and clinical signals, only a few cell therapy products have been approved globally, and most remain in development. However, substantial translational challenges remain, including variability in cell source and potency, limited persistence in joint environment, small clinical trial sizes, and regulatory and manufacturing hurdles. To achieve broader adoption, it will be essential to demonstrate superiority to minimally manipulated orthobiologics, clarify redosing strategies, and generate robust long-term evidence. This Review discusses recent clinical trial data, mechanistic insights, regulatory considerations, and operational challenges shaping the evolving role of cell therapy products for OA as next-generation candidates to bridge the gap between pharmacological and surgical interventions. In addition, this Review is written to support regulatory agencies as well as academics and clinicians involved in the development and evaluation of cell therapy products.

## Cell therapy products show promise for osteoarthritis relief

Osteoarthritis is a leading cause of pain and disability, affecting more than 600 million adults globally, with its prevalence rising due to aging and obesity. This Review explores the potential of cell-based orthobiological regenerative therapies, particularly mesenchymal stem cells (MSCs), which may provide durable benefits by modulating inflammation and supporting endogenous tissue repair. MSCs, despite rapid clearance from the knee joint, exhibit multifactorial mechanisms, including immunomodulation and chondroprotection, which could offer broader therapeutic effects than currently available treatments. However, clinical evidence for their superiority remains limited, highlighting the need for further mechanistic studies and stratified clinical trials. This Review emphasizes the importance of developing standardized manufacturing processes and regulatory frameworks to advance these therapies. Future directions include exploring cell-free approaches and enhancing MSC durability in the osteoarthritis environment, aiming for long-term clinical benefits and potential disease modification.

This summary was initially drafted using artificial intelligence, then revised and fact-checked by the author.

## Introduction

**Fig. 1:**
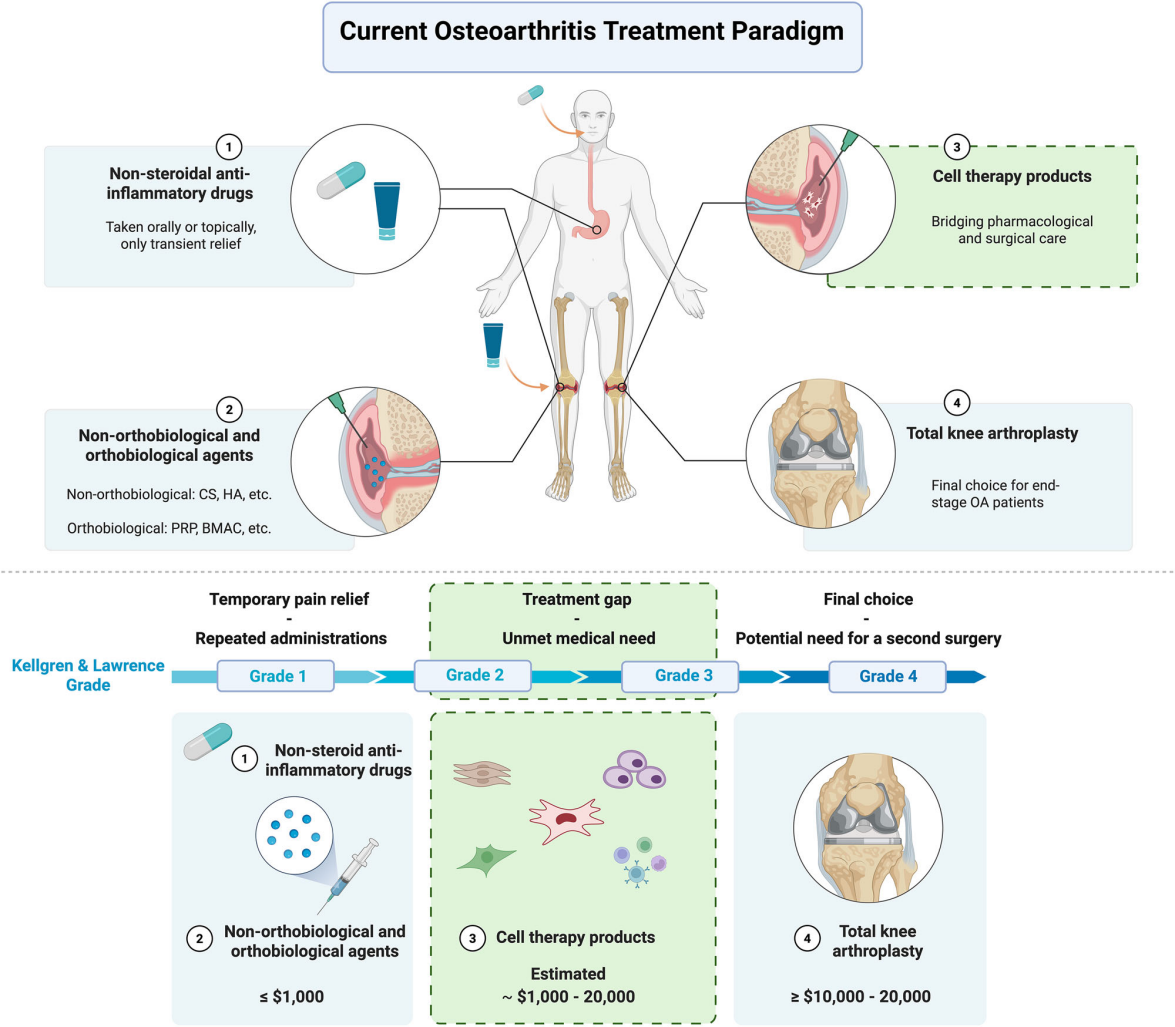
Current osteoarthritis treatment paradigm. Current osteoarthritis (OA) treatment paradigm consists of: (1) non-steroidal anti-inflammatory drugs administered orally or topically during the early stages of OA; (2) non-orthobiological intra-articular injections such as corticosteroids (CSs) or hyaluronic acid (HA), and orthobiological injections including platelet-rich plasma (PRP) or bone marrow aspirate concentrate (BMAC), also primarily used in early stages of OA; (3) prospective cell therapy products, mainly delivered through intra-articular injection, intended to bridge the therapeutic gap between early-stage and late-stage OA; (4) total knee arthroplasty as the final intervention when disease progression continues despite the aforementioned treatments. Figure created with BioRender.

Osteoarthritis (OA) affects more than 600 million adults globally and is among the leading causes of disability worldwide^1,2^. As the prevalence of OA continues to rise owing to aging and obesity, the burden on health-care systems grows correspondingly. Current OA treatment paradigms rely heavily on non-orthobiological symptom-modifying agents including non-steroidal anti-inflammatory drugs, corticosteroids (CS), and hyaluronic acid (HA), as well as orthobiologics, referred here as minimally manipulated autologous products, such as platelet-rich plasma (PRP) and bone marrow aspirate concentrate (BMAC)^3–6^ (Fig. 1). Although these approaches may provide transient relief based on patient characteristics and severity of OA, they do not modify the underlying disease progression^4,6,7^. In fact, repeated CS injections are associated with an increased risk of chondrotoxicity^6^. As a result, patients often experience years of inadequate pain control and functional limitation before progressing to surgical eligibility. Although total knee arthroplasty (TKA) is an effective intervention for end-stage OA, the treatment gap for patients with moderate disease remains unaddressed^8,9^. These patients are often symptomatic yet are not suitable candidates for surgical intervention and have few-to-no durable therapeutic options, highlighting the critical need for effective therapies after the aforementioned ones have failed; or if disease-modifying, the potential to treat patients earlier in the OA course (Fig. 1). Cell-based orthobiological regenerative therapy products hold promise in this regard by providing potentially durable, regenerative benefits, altering disease trajectory and improving long-term outcomes earlier in the OA patient journey (Boxes 1 and 2).

**Fig. 2:**
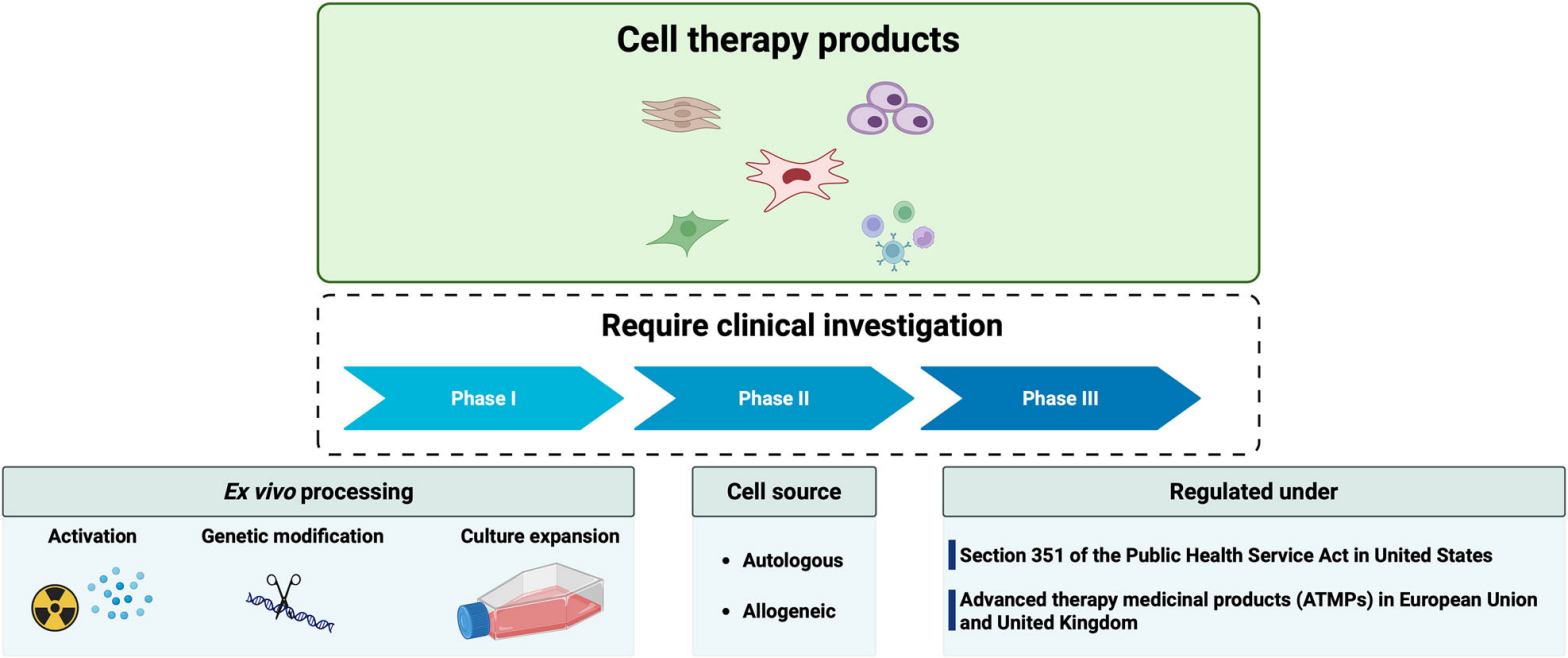
Regulatory overview of cell therapy products. Autologous or allogeneic cell therapy products require clinical investigation when they undergo substantial manipulation or ex vivo processing, such as activation with irradiation or cytokines, genetic modification, or culture expansion. In the USA, these products are regulated as Section 351 biologics under the Public Health Service Act, whereas in the European Union and the UK they are classified as ATMPs. Figure created with BioRender.

In this Review, we use the term “cell therapy products”^10^ to describe cell-based interventions that involve substantial ex vivo processing including culture expansion, activation, or genetic modification such that the resulting product requires formal regulatory approval as a biologic, for example, under Section 351 of the Public Health Service Act in the USA^11,12^ (Fig. 2). Within the European Union, these products fall under the category of advanced therapy medicinal products (ATMPs) and are regulated under the ATMP Regulation (EC) No. 1394/2007 and the associated Directive 2009/120/EC. Additional scientific guidance for investigational ATMPs and OA-relevant clinical development is provided by the EMA^13–15^. In the UK, ATMPs are regulated through the Medicines and Healthcare products Regulatory Agency under the ATMP regulatory and licensing guidelines^16^. This distinguishes cell therapy products from minimally manipulated, same-day, point-of-care autologous preparations such as PRP, BMAC, and stromal vascular fraction, which are regulated under less stringent pathways (for example, Section 361) and are not the focus of this Review.

Cell therapy products, particularly mesenchymal stem cells (MSCs), are emerging owing to their multifactorial mechanisms of action (MoA). Although most cells are rapidly cleared from the inflammatory knee OA environment after administration, they possess the potential to modulate regenerative effects through their multimodal MoA^17–19^. When compared with current anti-inflammatory and immunomodulatory treatments, cell therapy products in general exhibit a broader and more integrated profile of activity. Targeted biologics, such as anti-tumor necrosis factor alpha (TNF-α) or anti-interleukin-1 (IL-1) agents, and small molecules such as Janus kinase inhibitors, act through defined, single, or dual molecular targets. Although these agents have demonstrated efficacy in inflammatory arthritides such as rheumatoid arthritis^20,21^, their utility in OA remains limited, largely because OA is a mechanically and biologically heterogeneous disease not driven by a single inflammatory pathway. By contrast, cell therapy products, particularly MSCs, operate through multiple simultaneous mechanisms including: (i) immunomodulation via secretion of anti-inflammatory cytokines (IL-10), transforming growth factor-beta (TGF-β), IL-1 receptor antagonist (IL-1Ra), and attenuation of pro-inflammatory mediators (TNF-α, IL-1β, IL-6, matrix metalloproteinase 13 (MMP13), a disintegrin and metalloproteinase with thrombospondin motifs 4/5 (ADAMTS4/5))^22–24^; (ii) macrophage polarization from a pro-inflammatory M1 mediator toward an anti-inflammatory M2 phenotype within the synovium^22,25^; (iii) chondroprotective and anti-senescence effects through extracellular vesicle (EV)-mediated signaling and mitochondrial transfer^26–29^; and (iv) trophic support of endogenous repair through growth factor secretion. This multimodal activity profile, which acts across multiple cell types and compartments simultaneously, represents a broader mechanistic footprint than single-target agents, although whether this breadth translates into superior clinical outcomes remains unproven. Importantly, however, the clinical evidence that this broader mechanistic footprint translates into superior or additive outcomes over targeted agents in OA populations specifically remains limited and has not yet been tested in head-to-head clinical trials^30,31^. This remains a critical evidence gap that future clinical development should aim to address.

More than 960 non-genetically modified cell-based therapies are currently in development globally for a broad range of therapeutic indications^32^. Approximately 50 of these are being investigated for musculoskeletal conditions. When focusing on OA and culture-expanded manufacturing, it narrows the group to approximately 30 programs. Relatively small amounts of the initiated phase I studies finally advance to phase III, highlighting that promising outcomes in preclinical models remain challenging in clinical translation^33^, yet also reflecting the early stage of development in the cellular therapeutic product area for OA. To date, no cell therapy products for OA have received regulatory approval in the European Union, UK, or the USA, according to publicly available regulatory databases at the time of manuscript acceptance. However, four such manipulated agents have been approved in other international markets. Additionally, an allogeneic cell therapy product derived from equine umbilical cord has been approved in the UK and European Union for canine and equine OA^34–37^.

Our objective is to evaluate cell therapy products that involve substantial manipulation (for example, culture expansion, activation, and genetic modification) and therefore require formal clinical investigation and regulatory approval pathways. We summarize the underlying biology, clinical evidence, regulatory distinctions, and operational considerations that are shaping the future landscape of cell therapy products, with a particular emphasis on MSC-based interventions for OA.

Box 1 Regulatory expectations for cell therapy products **European and American regulatory expectations increasingly focus on** ^12^^,^^13^ ^,157–159^ **:**

• Cell viability assays

• Correlation between viable cell count and clinical effect

• Defined minimum cell viability thresholds

• Tight ranges for total cell count and viable cell count

• Implications of non-viable cells (for example, immunogenicity)

• Long-term safety monitoring (2–5 years)

• Local administration with systemic surveillance

• Cell persistence versus transient action (for example, M2 macrophage polarization and stromal activation)

• Cell safety (for example, tumorigenicity, viral or microbial contamination from culturing)

Box 2 Emerging directions in cell therapy product development • Operational advances (room-temperature stability and good manufacturing practices)

• Stratification of mesenchymal stem cell potency to patient endotype (“mesenchymal stem cell fitness”) via cell assays

• Development of innovative vector technologies for safer, more efficacious, non-integrative, and longer-lasting effects in gene-modified cell therapy products

• Shifting toward truly regenerative approaches, using advanced bioengineered scaffolds combined with gene-modified cell therapy products

• Transitioning toward cell-free secretome and extracellular vesicle-based therapies

## Biology of cell therapy products in OA

Recent insights highlight that the therapeutic activity of cell therapy products in OA is mediated less by direct differentiation into chondrocytes and more through paracrine and immunomodulatory mechanisms^38^. MSCs, for example, secrete a diverse repertoire of cytokines, growth factors, and EVs that can influence the joint microenvironment. Importantly, local senescent chondrocytes and MSCs in OA joints contribute to OA progression through the senescence-associated secretory phenotype (SASP)^39–41^, which fuels inflammation and matrix degradation. Preclinical data suggest that MSCs derived from young and healthy donors, administered intra-articularly, may counteract these processes, whereas senescent MSCs may have limited therapeutic efficacy, underscoring the importance of cell source, as it relates to potency, as well as expansion capability^42^. Underscoring the importance of healthy, young allogeneic cells, they may offer theoretical or preclinical advantages compared with more senescent autologous MSCs from older patients, although these advantages have not yet been demonstrated in head-to-head clinical comparisons^43,44^.

### Chondrocytes and genetically modified packaging cells

Chondrocytes, the heterologous cell population composing the articular cartilage, have a central role in maintaining the extracellular matrix (ECM) through the balanced synthesis of proteoglycans and collagens^45^. In OA, chondrocytes undergo phenotypic changes characterized by hypertrophy, senescence, and increased production of catabolic enzymes such as MMPs and ADAMTS, which accelerate cartilage breakdown^46–49^.

Challenges in chondrocyte-based therapies remain, including limited scalability owing to their proliferative capacity in vitro and the risk of dedifferentiation during expansion^50,51^. Chondrocytes are highly sensitive to their mechanical and biochemical microenvironment, requiring carefully optimized scaffolds or carriers to preserve their phenotype after implantation or injection. These limitations underscore why chondrocyte-based therapies, despite being successful in cartilage defect treatment, are less widely explored in OA than MSC-based approaches which are known to retain higher proliferation and differentiation potential, making them more suitable for industrial scale-up and the development of commercial off-the-shelf joint regeneration therapies. However, the clinical relevance of prolonged in vitro culture for dedifferentiation of chondrocytes remains unclear. The common belief that cells must resemble the target tissue to exert therapeutic benefit likely reflects from the cell replacement paradigm, in which transplanted cells are thought to restore the function by directly replacing damaged cells, whereas current evidence supports predominantly paracrine mechanisms.

**Fig. 3:**
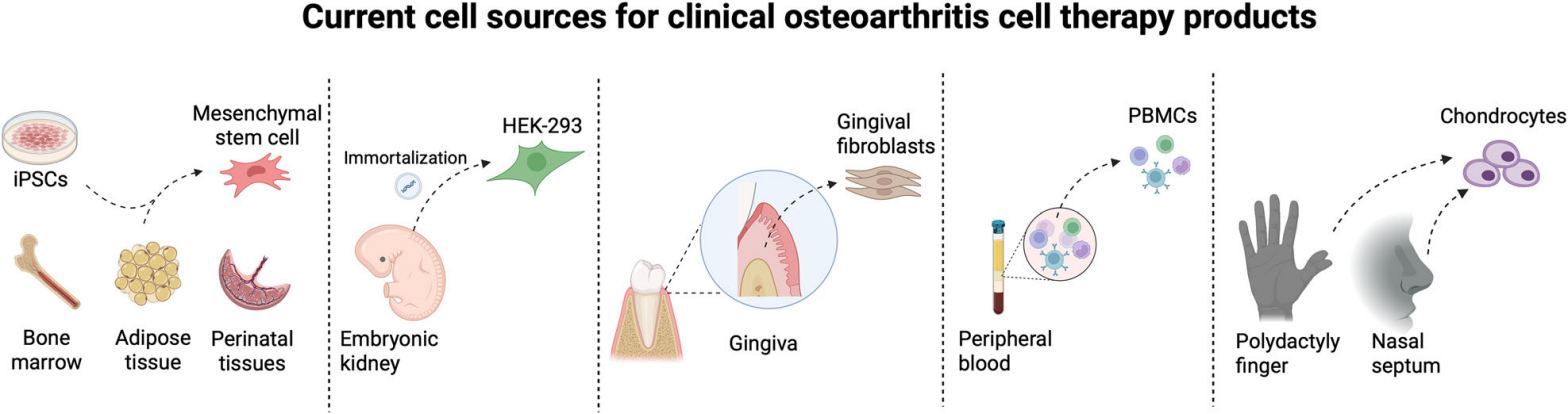
Current cell sources for clinical osteoarthritis cell therapy products. Mesenchymal stem cells (MSCs) are extracted from bone marrow, adipose tissue, and perinatal tissues. Additionally, MSCs are differentiated from induced pluripotent stem cells (iPSCs). Human embryonic kidney (HEK)-293 cell line is prepared by immortalizing primary embryonic kidney cell line. Stem cell-like gingival fibroblasts with regenerative capacity are extracted from adult gingiva. Peripheral blood mononuclear cells (PBMCs) can be extracted from peripheral blood. Chondrocytes are extracted from juvenile polydactyly fingers or nasal septum. Figure created with BioRender.

One promising off-the-shelf approach compared with MSCs involves the use of ex vivo culture-expanded chondrocytes derived from surgically removed polydactyly fingers (Fig. 3). These juvenile chondrocytes have been extensively studied for their potential in allogeneic cell therapy products for OA, including clinical cell-sheet transplantation^52,53^. Polydactyly-derived chondrocytes have superior proliferative profiles compared with autologous adult chondrocytes, maintain a stable chondrogenic phenotype during longer expansion periods, and demonstrate enhanced ECM production^50,51^.

Autologous culture-expanded chondrocytes from nasal septum offer better proliferatory and cartilaginous matrix-producing capabilities than the autologous articular cartilage chondrocytes^54–56^. Cartilaginous grafts have been prepared by culturing the nasal chondrocytes and implementing them in collagen type I/III membranes^54,57^. These cartilage grafts were utilized to treat chondral defects^54^, but later work has addressed patellofemoral OA^55^.

Another juvenile polydactyly-derived articular chondrocyte-based approach utilizes co-transplantation of chondrocytes with irradiated and transduced human embryonic kidney-293 (HEK-293)-derived GP2-293 cells engineered to produce TGF-β1 (refs. ^58,59^). HEK-293 cells are immortalized packaging cells derived by transforming primary embryonic kidney cells with a fragment of adenovirus type 5 DNA using calcium-phosphate-mediated transfection^60^. This gene-modified platform, unique among cell therapies, remains the only example of a clinically developed therapy based on genetically engineered cells. The 4.3 kb adenoviral fragment contains early region 1 (E1) transcription units E1A and E1B, integrated into chromosome 19 of the host genome^61,62^, enabling viral gene expression and therapeutic protein overexpression under viral, mammalian, or synthetic promoters.

### Mesenchymal stem cells

In 2006, the International Society for Cell and Gene Therapy (ISCT) proposed minimal criteria for MSCs (also referred to as mesenchymal stromal cells, or more controversially medicinal signaling cells^63^), based on their ability to adhere to plastic under standard culture conditions, expression of specific surface markers, and their capacity for trilineage differentiation potential into adipogenic, chondrogenic, and osteogenic lineages^64^. In 2016, ISCT expanded the guidelines by proposing additional assays defining MSC biology^65^. A subsequent update in 2019 highlighted the importance of the tissue of origin together with surface markers and functional assays^66^. MSCs have been studied since the 1960s^67^ and continue to show interest in the ongoing clinical trials. Even though they have the ability to differentiate into chondrocyte-like cells as shown in the early OA studies, their main MoA does not lie in replacement therapy^68,69^. Despite the low survival in the inflammatory OA environment, MSCs may still possess the ability to modulate OA disease progression through their therapeutic multimodal MoA^3,70,71^.

MSCs can be obtained from various sources, including adult tissues such as adipose tissue^72^, BM^67,73^, and dental tissues^74^, as well as perinatal tissues such as the placenta^75^, umbilical cord blood^76^, and Wharton’s jelly^77^. A major limitation for culture-expanded MSCs is their potency for hypertrophic differentiation^78,79^. MSCs derived from BM, adipose tissue, umbilical cord, placenta, amniotic fluid, and laboratory-induced pluripotent stem cells (iPSCs) are being developed in the clinic as cell therapy products of OA therapies (Fig. 3 and Tables 1 and 2).

**Table 1:** Culture-expanded cell therapy products in clinical development for osteoarthritis.

	Agent	Company/sponsor	Mechanism of action	Allogeneic versus autologous	Cell source	Number of cells in dose	Route of administration	Development stage/status	Year approved	Country of lead company	Clinical references
1	Cartistem (formerly SMUPIA01)	Medipost	Implanted allogeneic umbilical cord blood-derived mesenchymal stem cells with 4% hyaluronic acid	Allogeneic	Umbilical cord blood	2.5 × 10^6^ cells/500 µl/cm^2^	Arthrotomy or arthroscopy	Approved in South Korea	2012	South Korea	NCT01733186, NCT01626677, NCT01041001, NCT04234412
2	JointStem	R Bio Co. Ltd (Biostar Stem Cell Research Institute)	Autologous adipose-derived mesenchymal stem cells	Autologous	Adipose tissue	100 × 10^6^ cells	Intra-articular	Approved in Japan	2015	South Korea	NCT04368806, NCT02658344, NCT02674399, NCT03990805
3	MesestroCell	Cell Tech Pharmed	Autologous bone marrow-derived mesenchymal stem cells	Autologous	Bone marrow	20 × 10^6^ cells	Intra-articular	Approved in Iran	2018	Iran	NCT01207661, NCT01436058, NCT01499056
4	Stempeucel® (aka StemOne)	Stempeutics Research (distributed by Alkem, licensed to Cipla in India)	Allogeneic bone marrow-derived mesenchymal stem cells and 20 mg of hyaluronic acid	Allogeneic	Bone marrow	25 × 10^6^ cells	Intra-articular	Approved in India	2020	India	CTRI/2018/09/015785, NCT01453738, NCT01448434
5	AlloJoin	AbelZeta Pharma (formerly Cellular Biomedicine Group)	Allogeneic adipose-derived mesenchymal stem cells	Allogeneic	Adipose tissue	Unknown	Intra-articular	Phase III		China	NCT02641860, NCT04208646, NCT06570291
6	CLS2901C	Cellseed	Allogeneic polydactyly excised finger-derived cartilage	Allogeneic	Chondrocytes (polydactyly finger)	Unknown, chondrocyte sheets	Arthrotomy, proximal tibial osteotomy	Phase III		Japan	NCT06549686
7	CYP-004	Cynata Therapeutics	Allogeneic mesenchymal stem cells derived from mesenchymoangioblasts derived from induced pluripotent stem cells	Allogeneic	Peripheral blood, induced pluripotent stem cells	25 × 10^6^ cells	Intra-articular	Phase III		Australia	ACTRN12620000870954
8	Elixcyte	UnicoCell BioMed	Allogeneic adipose-derived mesenchymal stem cells	Allogeneic	Adipose tissue	32 × 10^6^ cells	Intra-articular	Phase III		Taiwan	NCT05526001, NCT02784964
9	IxCell hUC-MSC-O	Shanghai IxCell Biotechnology Co., Ltd	Allogeneic umbilical cord-derived mesenchymal stem cells	Allogeneic	Umbilical cord	50 × 10^6^ cells	Intra-articular	Phase III		China	NCT06716281
10	MAG200	Magellan Stem Cells	Allogeneic adipose-derived mesenchymal stem cells	Allogeneic	Adipose tissue	20 × 10^6^ or 100 × 10^6^ cells	Intra-articular	Phase III		Australia	NCT07106229, ACTRN12617001095358, ACTRN12621000622808
11	TG-C (formerly Invossa)	Kolon TissueGene (KTG)	Non-transduced human allogeneic polydactyly excised finger-derived primary articular chondrocytes and irradiated *TGF-β1* transgene transduced GP2-293 cells	Allogeneic	Chondrocytes (polydactyly finger), GP2-293 (Human embryonic kidney-293, embryonic kidney)	30 × 10^6^ cells	Intra-articular	Phase III		South Korea	NCT02341391, NCT02341378, NCT02072070, NCT01671072, NCT00599248, NCT01221441, NCT03291470, NCT05276011, NCT03203330
12	Adipose-derived mesenchymal stem cell	Montpellier University Hospital	Autologous adipose-derived mesenchymal stem cell	Autologous	Adipose tissue	2 × 10^6^ or 10 × 10^6^ cells	Intra-articular	Phase II		France	NCT01585857, NCT02838069
13	Chondrochymal®	Taiwan Bio Therapeutics	Allogeneic bone marrow-derived mesenchymal stem cells	Allogeneic	Bone marrow	50 × 10^6^ cells	Intra-articular	Phase II		Taiwan	NCT05027581, NCT03589287
14	iSCLife-UT	SCLnow Biotechnology	Allogeneic umbilical cord-derived mesenchymal stem cells	Allogeneic	Umbilical cord	10 × 10^6^ or 20 × 10^6^ cells	Intra-articular	Phase II		China	NCT03383081
15	N-TEC	University Hospital, Basel, Switzerland	Autologous cartilage graft composing of human nasal chondrocytes within a collagen type I/III membrane	Autologous	Nasal cartilage	50 × 10^6^ cells per 30 × 40 × 2 mm membrane	Arthrotomy	Phase II		Switzerland	NCT06576583, NCT06163573
16	SALIAI-UC1	Guangzhou SALIAI Stemcell	Allogeneic umbilical cord-derived mesenchymal stem cells	Allogeneic	Umbilical cord	50 × 10^6^ or 100 × 10^6^ cells	Intra-articular	Phase II		China	CTR2022201, CTR20252104
17	Umbilical cord-derived mesenchymal stem cells	Wuhan Hamilton Biotechnology Co., Ltd	Allogeneic umbilical cord-derived mesenchymal stem cells	Allogeneic	Umbilical cord	5 × 10^6^, 10 × 10^6^, or 20 × 10^6^ cells	Intra-articular	Phase II		China	NCT06463847, CTR20252299, CTR20240763
18	ZGHUMSC001	Guizhou Zhongguan Biotechnology Co., Ltd	Allogeneic umbilical cord-derived mesenchymal stem cells	Allogeneic	Umbilical cord	30 × 10^6^ or 50 × 10^6^ cells	Intra-articular	Phase II		China	CTR20222534, CTR20240818
19	Adipose-derived mesenchymal stem cells	Aarhus University Hospital	Allogeneic adipose-derived mesenchymal stem cells	Allogeneic	Adipose tissue	20 × 10^6^ cells	Intra-articular	Phase I/II		Denmark	NCT05933434
20	ANT-301 (aka ALLO-ASC-OA)	Anterogen Co., Ltd	Allogeneic adipose-derived mesenchymal stem cells and fibrin hydrogel	Allogeneic	Adipose tissue	Unknown	Intra-articular	Phase I/II		South Korea	NCT06539429
21	Cellistem®-OA	CellTech Pharmed	Allogeneic umbilical cord tissue-derived mesenchymal stem cells (primarily Wharton’s jelly)	Allogeneic	Umbilical cord	2 × 10^6^ cells	Intra-articular	Phase I/II		Iran	NCT04863183, NCT03810521, NCT06431152 Exosomes, NCT05060107 Exosomes
22	Furestem-OA Kit	Kangstem Biotech	Allogeneic umbilical cord blood-derived mesenchymal stem cells	Allogeneic	Umbilical cord blood	Unknown	Intra-articular	Phase I/II		South Korea	NCT05944627, NCT06013306
23	PLX-PAD (formerly Emiplacel)	Pluri Biotech Ltd (formerly Pluristem Therapeutics)	Allogeneic placental-derived mesenchymal-like adherent stromal cells	Allogeneic	Placental	Unknown	Intra-articular, periarticular muscle	Phase I/II		Israel	2023-504212-15-00
24	StromaForte	Cellcolabs Clinical Ltd	Allogeneic bone marrow-derived mesenchymal stem cells	Allogeneic	Bone marrow	50 × 10^6^ cells	Intra-articular	Phase I/II		The Bahamas	NCT06084988
25	Xinbang mesenchymal stem cell injection	Shandong Xingrui Biotech	Allogeneic adipose-derived mesenchymal stem cells	Allogeneic	Adipose tissue	10 × 10^6^, 20 × 10^6^, 40 × 10^6^, or 50 × 10^6^ cells	Intra-articular	Phase I/II		China	CTR20233065
26	XSTEM-OA	Xindu Pty Ltd (Xintela)	Allogeneic adipose-derived mesenchymal stem cells	Allogeneic	Adipose tissue	Unknown	Intra-articular	Phase I/II		Australia	NCT05344157
27	Allogeneic engineered gingival fibroblasts (aeGFs)	Scarcell Therapeutics S.A.S	Allogeneic engineered gingival fibroblasts	Allogeneic	Gingival fibroblasts	50 × 10^6^ cells	Intra-articular	Phase I		France	NCT06690710
28	Amniotic fluid mesenchymal stem cells developed for chondrogenic treatment (AFCC)	Siriraj Hospital at Mahidol University, Thailand	Allogeneic amniotic fluid-derived mesenchymal stem cells	Allogeneic	Amniotic fluid	20 × 10^6^ cells	Intra-articular	Phase I		Thailand	NCT06386679
29	Autologous mesenchymal stem cell therapy	Chinese University of Hong Kong	Autologous bone marrow-derived mesenchymal stem cells	Autologous	Bone marrow	6 × 10^6^cells	Intra-articular	Phase I		Hong Kong	CUHK_CCT00469
30	Human adipose-derived mesenchymal stem cell injection	Bopin Biomed (Shanghai/Shenzhen)	Allogeneic adipose-derived mesenchymal stem cells	Allogeneic	Adipose tissue	10 × 10^6^, 20 × 10^6^, or 50 × 10^6^ cells	Intra-articular	Phase I		China	CTR20223422
31	Human umbilical cord mesenchymal stem cells	First Affiliated Hospital of Wannan Medical College	Allogeneic umbilical cord tissue-derived mesenchymal stem cells	Allogeneic	Umbilical cord	25 × 10^6^ cells	Intra-articular	Phase I		China	NCT06082440
32	Progenza	Regeneus/Cambium Bio Ltd	Allogeneic adipose-derived mesenchymal stem cells	Allogeneic	Adipose tissue	3.9 × 10^6^cells or 6.7 × 10^6^cells	Intra-articular	Phase I		Australia	ACTRN12615000439549
33	UMC119-06-05	Meridigen Biotech Co., Ltd	Human umbilical cord-derived mesenchymal stem cells with hyaluronic acid	Allogeneic	Umbilical cord	Unknown	Intra-articular	Phase I		Taiwan	NCT04893174

**Table 2:** Non-expanded intra-articularly administered cell therapy products in clinical development for osteoarthritis.

	Agent	Company/sponsor	Mechanism of action	Allogeneic versus autologous	Cell source	Number of cells in dose	Development stage/status	Culture propagation of stem cells	Country of lead company	Clinical references
1	GID BIO SVF-2	GID BIO, Inc.	Autologous adipose-derived stromal vascular fraction	Autologous	Adipose tissue	Unknown	Phase III	Non-expanded/manipulated	USA	NCT02726945, NCT04440189
2	MFat	Lipogems	Autologous microfragmented adipose tissue	Autologous	Adipose tissue	Unknown	Phase III	Non-expanded/minimally manipulated (Section 361)	Italy	NCT03922490, NCT06121882, NCT05660772, NCT03714659
3	Transpose® RT System combined with Matrase	InGeneron	Autologous adipose-derived mesenchymal stem cells	Autologous	Adipose tissue	Unknown	Phase III	Non-expanded/manipulated	USA	NCT03503305, NCT04405297, NCT03513731
4	Allocetra, Allocetra-OTS, OTS-Allocetra	Enlivex Therapeutics	Irradiated allogeneic peripheral blood mononuclear cells (PBMCs) induced to apoptosis	Allogeneic	Peripheral blood	24.3 × 10^6^ or 49.5 × 10^6^ cells	Phase II	Non-expanded/manipulated	Israel	NCT06233474, NCT06208241, NCT06459063 (thumb joint osteoarthritis (OA))
5	OA-SYS	Ageless Biotech	Autologous adipose-derived stromal vascular fraction	Autologous	Adipose tissue	Unknown	Phase II	Non-expanded/minimally manipulated (Section 361)	USA	NCT06485843
6	StroMel™	Akan Biosciences LLC	Autologous adipose-derived mesenchymal stem cells	Autologous	Adipose tissue	Unknown	Phase II	Non-expanded/minimally manipulated (Section 361)	USA	NCT04750252
7	GeneXSTEM	BioIntegrate LLC	Allogeneic micronized umbilical cord tissue	Allogeneic	Umbilical cord	Unknown	Phase I/II	Non-expanded/minimally manipulated (Section 361)	USA	NCT05234489
8	Signature Cord Prime™ (SIG001)	Signature Biologics	Allogeneic micronized umbilical cord tissue	Allogeneic	Umbilical cord	75 mg or 150 mg diluted in 4 ml of Ringer’s lactate	Phase I	Non-expanded/minimally manipulated (Section 361)	USA	NCT05234489

The emergence of off-the-shelf allogeneic MSC products aims to reduce variability while enabling scalable and standardized solutions for broad clinical applications. By contrast, autologous expanded MSC therapies, although considered the safest owing to their immunocompatibility, are labor-intensive, time-consuming, show a high variability between products, and represent the most expensive option. Although iPSC-derived therapies, which have been under investigation since their discovery in 2006 (ref. ^80^), are promising candidates, they still face substantial barriers to clinical translation, including limited standardization, predictability, reproducibility, and regulatory challenges.

### Therapeutic mechanism of action

The therapeutic effects of MSCs fall within several macro-areas, grouped as follows.

#### Immunomodulation and paracrine signalling

**Fig. 4:**
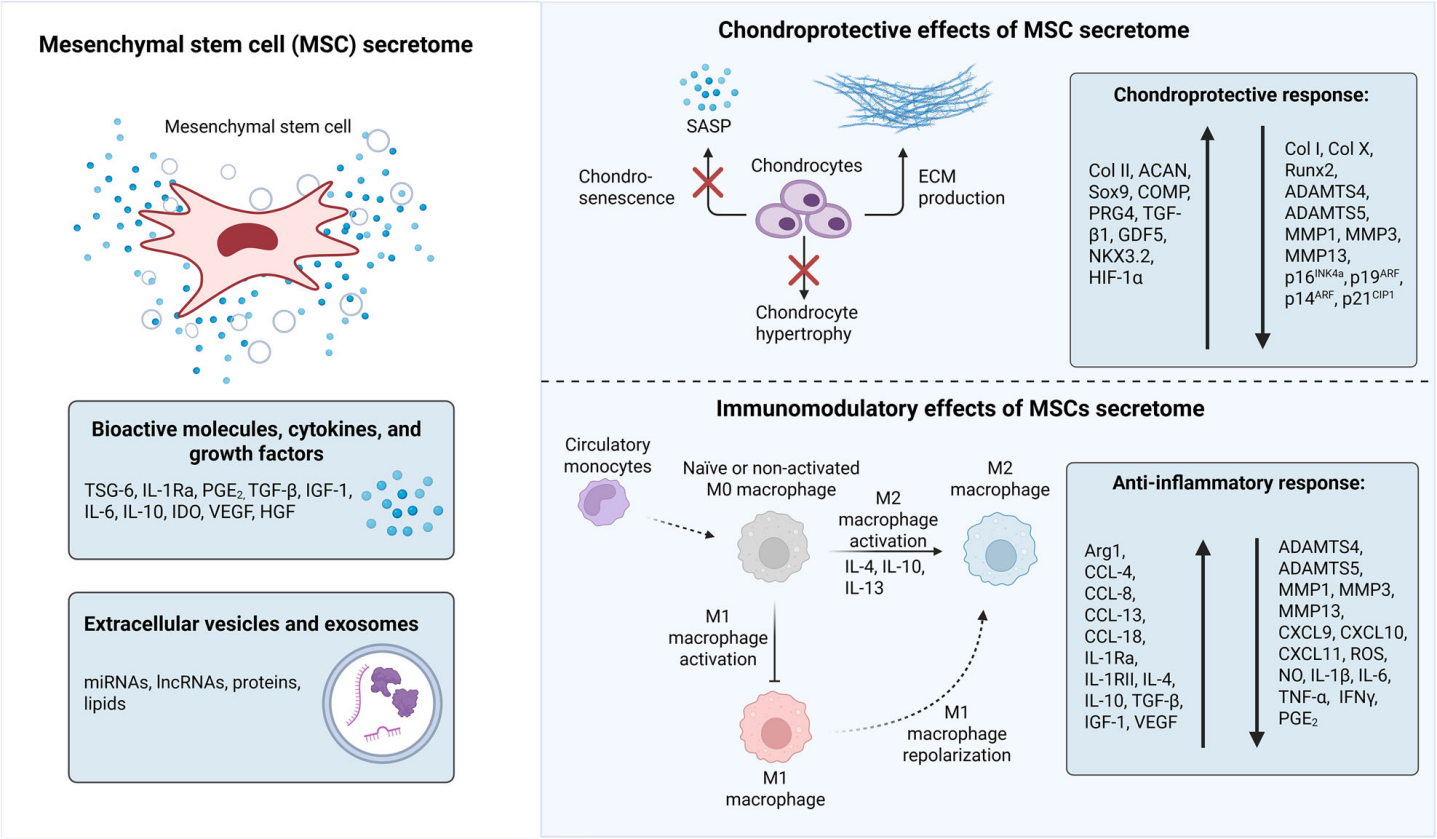
MSCs have multimodal effects mainly through their secretome. MSCs can secrete bioactive molecules, cytokines, growth factors, extracellular vesicles (EVs), and exosomes related to osteoarthritis (OA) disease-modifying mechanisms. MSC secretome can especially have an effect on chondrocytes by decreasing cellular senescence, chondrocyte hypertrophy, and by inducing cartilaginous extracellular matrix (ECM) production. The secretome can also have immunomodulatory effects by inducing anti-inflammatory response by the immune cells, and increase the polarization of macrophages to anti-inflammatory M2 macrophage lineage. ACAN aggrecan, ADAMTS a disintegrin and metalloproteinase with thrombospondin motifs, Arg1 arginase 1, CCL-4, -8, -13, -18, C-C motif chemokine ligands; Col I, Col II, Col X, collagen type I, type II, type X; COMP cartilage oligomeric matrix protein, CXCL C-X-C motif chemokine ligands, GDF5 growth differentiation factor 5, HGF hepatocyte growth factor, HIF-1α hypoxia-inducible factor 1-alpha, IDO indoleamine 2,3-dioxygenase, IFN-γ interferon gamma, IGF-1 insulin-like growth factor 1, IL-1β interleukin-1 beta, IL-1RII interleukin-1 receptor type II, IL-1Ra interleukin-1 receptor antagonist, IL-4, -6, -10, -13, interleukins; lncRNA long non-coding RNA, miRNA microRNA, MMP matrix metalloproteinase, NKX3.2 NK3 homeobox 2, NO nitric oxide, p14^ARF^, p16^INK4a^, p19^ARF^ p21^CIP1^, senescence markers; PGE_2_ prostaglandin E_2_, PRG4 proteoglycan 4, Runx2 runt-related transcription factor 2, SASP senescence-associated secretory phenotype, TGF-β transforming growth factor-beta, TNF-α tumor necrosis factor-alpha, TSG-6 tumor necrosis factor-stimulated gene 6, VEGF vascular endothelial growth factor. Figure created with BioRender.

MSC secretome and especially EVs have shown protective effects in OA joints by promoting proliferative and anti-apoptotic effects^81^. The EVs contain microRNAs, long non-coding RNAs, and immunoregulatory proteins, targeting the immune landscape^27,82,83^ (Fig. 4). EV contents can act on chondrocytes, promoting the expression of cartilage-specific ECM genes, while reducing expression of catabolic enzymes, triggering autophagy^84^, and weaken ferroptosis^85,86^. Cell-free therapies are now recognized as mediators of MSC effects, and they provide a rationale for the development of exosome-based or secretome-based therapies as next-generation approaches.

The main therapeutic MoA linked to MSCs is their immunomodulatory function^87^, mediated by reducing the release of pro-inflammatory cytokines and enzymes including TNF-α, IL-1β, IL-6, MMP1, MMP3, MMP13, ADAMTS4, ADAMTS5 (refs. ^24,88–91^). These factors are secreted by chondrocytes in the articular cartilage, synovial fibroblasts, synoviocytes, and resident macrophages within the synovial membrane and synovium^22,92–95^. MSCs can have the ability to modify circulatory monocytes naive or non-activated M0 macrophages, and less commonly, induce pro-inflammatory M1 macrophage phenotype repolarization in the synovium toward an anti-inflammatory M2 macrophage phenotype^25,81,96,97^ (Fig. 4). These actions can promote an anti-inflammatory phenotype in the synovium by enhancing the production of cytokines and growth factors, such as IL-4, IL-10, and TGF-β^19,23,26,98^. Another key immunomodulatory factor secreted by MSCs is IL-1Ra, which inhibits the binding of IL-1 to its receptor, and suppresses downstream IL-1-mediated signaling^70,99,100^.

#### Chondroprotective and neuromodulatory effects

All the aforementioned factors contribute to chondroprotective effects by preventing chondrocyte apoptosis, enhancing ECM production, decreasing chondrocyte hypertrophy, and weaken the chondrocyte senescence phenotype displayed by decreasing the expression of SASPs^26,40,45,101,102^. Most recently, such features were achieved through MSC mitochondrial transfer to chondrocytes by reducing oxidative stress and restoring energy metabolism^28,29,103,104^. MSCs can promote chondrocyte activity by inhibiting their differentiation into fibroblast-like phenotype, thereby reducing the fibrocartilage ECM production and fibrosis^105^.

Recent clinical studies highlight the limited evidence supporting direct pain-modulatory mechanisms of MSC therapies^31^. Nonetheless, MSCs have been reported to alleviate pain through multiple mechanisms^22^. In preclinical models, they directly interact with pain mediators such as substance P and calcitonin gene-related peptide, leading to reduced nociceptive signalling^22,106,107^. Additionally, MSCs can modulate pain indirectly through the inflammatory environment in synovium by reducing production of pro-inflammatory cytokines such as TNF-α, IL-6, and IL-1β while promoting anti-inflammatory pathways^22^.

### Other cell sources

Fibroblasts can be extracted from various tissues, as they are the main cells producing ECM. For OA therapies, fibroblasts having multi-differentiation potential have been modified and extracted from gingiva (NCT06690710)^108^ (Fig. 3). Gingival fibroblasts have been proposed to have similar multimodal and immunomodulatory behavior as MSCs^109^.

Peripheral blood mononuclear cells (PBMCs) can be extracted from the circulating bloodstream using centrifugation and collecting the lymphocyte layer^110^. For OA therapeutics, allogeneic PBMCs have been cultured, irradiated, and cryopreserved (NCT06233474)^111,112^. They have been proposed to modify the immunomodulatory response in the knee OA.

## Current clinical development landscape

The majority of the clinical trials investigating cell therapy products for OA measure pain reduction with Visual Analogue Scale (VAS) and evaluate functional improvements mainly through the Western Ontario and McMaster Universities Osteoarthritis Index (WOMAC) or the International Knee Documentation Committee (IKDC) score. The Knee injury and Osteoarthritis Outcome Score (KOOS) also evaluates both pain and functional improvements, and these clinical assessments are usually complemented by imaging-based markers using MRI. However, clear clinical evidence demonstrating that cell therapy products can modify synovial macrophage polarization, reduction of pro-inflammatory cytokines, or secretome-mediated effects relies on the extensive in vitro and in vivo translational work performed with animal models and in laboratories. This means that such mechanisms cannot be convincingly demonstrated in human OA joints so far. Addressing these limitations would require alternative clinical trial designs.

Within the framework of recent clinical trial designs, there is no clear clinical evidence that young allogeneic MSCs, despite potentially having better cellular proliferative, senescence, or apoptotic profiles, produce a better biological response in the human OA environment than autologous MSCs. At this point, there is only preclinical evidence to support age-related effects on MSC fitness and their potential therapeutic implications^113,114^. The biological plausibility of this argument derives from well-characterized phenomena, including donor-age-related MSC senescence, reduced paracrine secretory capacity, and diminished immunomodulatory potency, that have been demonstrated in cell culture and animal models. However, direct clinical head-to-head comparisons between allogeneic and autologous MSCs in OA populations are currently absent from the published literature. Two recent meta-analyses reviewed five and eight randomized controlled trials (RCTs) involving culture-expanded intra-articularly injected MSCs for knee OA^31,115^. Both included allogeneic and autologous programs, but no study has been designed to isolate donor age as an independent variable. As a result, any assertion of clinical superiority of allogeneic MSCs over autologous MSCs based on donor age remains a hypothesis that requires prospective clinical testing.

Within the two meta-analyses reviewed, most trials showed improvements in WOMAC, KOOS, VAS, and MRI measures in the MSC-treated arms compared with controls^31,115^. However, heterogeneity in cell source, dose, and study design complicates direct comparisons, but adipose-derived (AD) MSCs showed more efficiency^115^. These results are contradictory to a recent phase IIb clinical trial utilizing autologous culture-expanded AD-MSCs showing no significant improvement in pain and function^30^. Higher-dose arms, commonly in the 40–50 million-cell range, were often associated with greater improvement, although this pattern was not universal and should not be interpreted as a validated dose-response relationship^31,115^. Kellgren–Lawrence (KL) grades 2–3 were the most frequently studied populations. Patient-specific factors such as baseline pain severity and OA phenotype influenced the therapy responses. Cryopreservation method, donor source (single versus pooled), and co-injection with biologics (for example, PRP and HA) also contributed to variability in outcomes^31^. The main caveat for the RCTs reviewed was the lack of follow-up time.

Although the administered cells are believed to clear rapidly from the OA joint environment typically within days to weeks, as supported by preclinical tracking studies, the positive results observed in several clinical studies are not necessarily paradoxical. Durable benefit in the absence of long-term cell persistence is consistent with a “hit-and-run“ or “kickstart“ model of action, in which the therapeutic window of cell activity is sufficient to initiate downstream biological cascades that outlast the cells themselves^116^. Several non-mutually exclusive mechanisms may account for this dissociation: (i) macrophage polarization; (ii) reprogramming of resident joint cells; (iii) amplification of endogenous repair pathways; and (iv) neuromodulatory effects.

It is important to note that although these mechanisms are well supported in preclinical models, their relative contribution to durable clinical effects in human OA joints has not been directly demonstrated and remains an important unresolved question. Future trial designs incorporating mechanistic biomarkers, synovial biopsies, and longitudinal imaging will be essential to confirm whether microenvironmental reprogramming is indeed the principal driver of sustained clinical benefit in patients with cell therapy-treated OA. These mechanisms are largely derived from preclinical and in vitro systems, and their magnitude and durability in human OA joints remain uncertain. Alternative explanations, including natural fluctuation of symptoms and placebo effects, must also be considered when interpreting durability signals in small trials.

Adverse events (AEs) associated with cell therapy products for OA are typically mild and transient, including pain and swelling at the injection site, and joint discomfort^3,117,118^. Serious AEs are rare and often due to underlying comorbidities unrelated to therapy, particularly in studies in which patients are not screened for chronic diseases before enrollment^31^.

### Prominent clinical programs

**Fig. 5:**
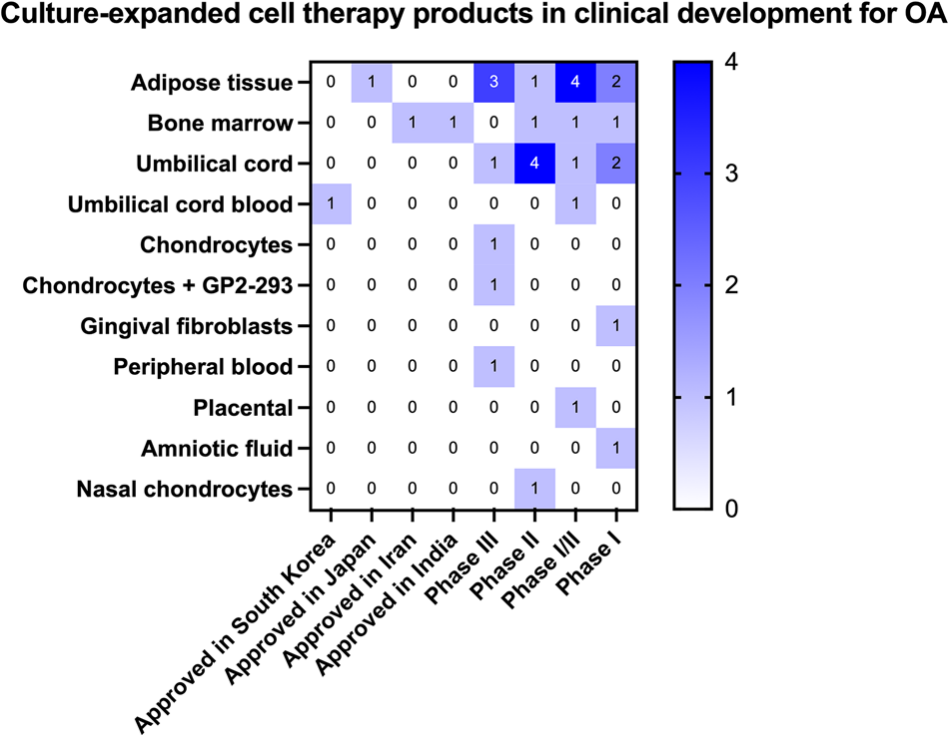
Culture-expanded cell therapy products in clinical development for osteoarthritis. Figure created using GraphPad Prism.

**Fig. 6:**
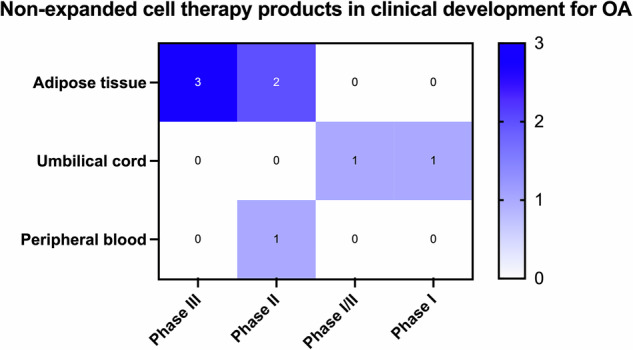
Non-expanded cell therapy products in clinical development for osteoarthritis. Figure created using GraphPad Prism.

From the approximately 40 clinical candidates (Figs. 5 and 6), there are six prominent programs, of which five are administered with intra-articular injections and one is administered surgically (for example, arthrotomy).

Cartistem from Medipost requires a surgical arthrotomy implantation of allogeneic human umbilical cord blood-derived MSCs targeting mainly International Cartilage Repair Society (ICRS) grade 3–4 patients with OA^119–126^. In the procedure, multiple holes are drilled into the subchondral bone of the defected areas and the human umbilical cord blood-derived MSCs, accompanied by 4% HA, are injected into the drilled holes. A 7-year phase I/II follow-up safety and efficacy trial of the product with seven participants was evaluated with no serious AEs, with improved and sustained IKDC and VAS scores^122^. In a randomized phase III trial conducted in South Korea for ICRS grade 4 patients, Cartistem showed improvements in IKDC, VAS, WOMAC, and ICRS scores up to 3–5 years of follow-up, improving pain and function of aged patients^121^. Cartistem is anticipated to enter phase III trials in the USA in 2026 after finishing phase II (NCT01733186)^123^. The follow-up from phase III dosing is ongoing in Japan.

Stempeucel from Stempeutics is an allogeneic, culture-expanded, pooled, BM-derived MSC therapy designed for KL grade 2–3 patients through a single intra-articular injection of 25 million cells into the knee joint, followed by 20 mg of HA injection^127–130^. Stempeucel as pooled BM-MSCs have been demonstrated to induce multipotency of the cells in vitro compared with single donor MSCs^131^. In August 2020, the product was authorized for manufacturing and marketing in India by the Drug Controller General of India. A 60-patient phase II (NCT01453738) escalation study demonstrated safety of the product with a low-dose group, but reported similar results compared with the HA-treated placebo group showing no superiority of the treatment^129^. In a 146-patient randomized phase III (CTRI/2018/09/015785) study, pooled Stempeucel from three healthy donors demonstrated efficacy with improved WOMAC scores at both 6 and 12 months, with no evidence of disease progression as assessed by MRI compared with HA-treated placebo group^128^. The product is cryopreserved as 25 million pooled BM-MSCs with more than 20 million cell viability in 1 ml of CryoStor CS5 Crystal Zenith vials^128^.

Progenza from Regeneus utilizes culture-expanded allogeneic AD-MSCs, targeting KL grade 1–3 patients. A 20-patient phase I study (ACTRN12615000439549) with intra-articular injections of low (3.9 × 10^6^ cells, *n* = 8) and high (6.7 × 10^6^ cells, *n* = 8) doses of AD-MSCs demonstrated improvements in both VAS and WOMAC scoring at months 3, 6, 9, and 12 (ref. ^132^). The administered number of cells in the dose is among the minor end of the therapies reviewed reporting the number of cells.

Tissuegene-C (TG-C) from Kolon TissueGene currently stands out as the only gene-modified cell-based therapy in clinical development for KL grade 2–3 OA with two 530 patient phase III trials (NCT03203330 and NCT03291470)^58,59,133^. The MoA involves GP2-293 cells, a HEK-293 derivative cell line engineered to stably express the *gag* and *pol* genes but lack the *env* gene. This enables the production of retroviral particles when co-transfected with appropriate envelope-coding gene, facilitating retroviral gene delivery. These cells are stably transduced with a plasmid encoding the human *TGF-b1* transgene and then γ-irradiated to inhibit cellular proliferation. The modified GP2-293 cells are mixed in a 1:3 ratio with primary articular cartilage chondrocytes derived from the polydactyly finger of a 1-year-old female donor^134^, and 2 ml (30 × 10^6^ cells/2 ml) of the final mixture is administered via intra-articular injection. TG-C has been suggested to have potential analgesic and immunomodulatory effects in the rat monoiodoacetate-induced model of OA^59,133^. The in vivo immunomodulatory effects were further investigated by inducing M2 macrophage polarization in the in vitro THP-1 cell line. The findings proposed that the immunomodulation is achieved mainly through prostaglandin E_2_ signaling induced by TGF-β1 production^58,133^.

Allocetra from Enlivex Therapeutics is the only off-the-shelf allogeneic mononuclear cell therapy product targeting KL grade 2–3 patients in clinical trials (phase I/II, NCT06233474). The therapy utilizes apoptosis-induced PBMCs, triggering immunomodulatory responses by interacting with innate immune cells such as macrophages^112,135^. The PBMCs are collected from a healthy donor using apheresis, cultured with medium containing methylprednisolone, resuspended, irradiated (X-ray, 4,000 cGy), and cryopreserved^135^. Intra-articular injections of cryopreserved, apoptosis-induced, and irradiated PBMCs have shown clinical and non-clinical safety^112,136^.

PLX-PAD from Pluri Biotech Ltd is an allogeneic culture-expanded placental-derived MSC-like therapy shown to be safe in two phase III trials for critical limb ischemia (NCT03006770) and regeneration of muscle injury following surgery for hip fracture arthroplasty (NCT03451916), currently in a combined phase I and IIa trial (2023-504212-15-00) with follow-up of 3 years for the treatment of KL grade 2–3 OA. The preclinical safety of the treatment has been demonstrated through intra-articular injections to Dunkin Hartley guinea pigs with knee OA^137^. The proposed MoA is mediated by cytokines, chemokines, and growth factors secreted by the PLX-PAD cells, which modulate innate and immune cell proliferation and promote the production of anti-inflammatory factors^138^.

## Clinical product landscape: approved and late-phase therapies

Real-world clinical experience is emerging for a handful of approved MSC-based products.

These clinical products illustrate diversity in cell source, geographic regulatory pathways, and clinical positioning (Tables 1 and 2). Cartistem and Stempeucel are the only allogeneic models approved, offering scalable off-the-shelf use. JointStem and MesestroCell are autologous, with higher logistical complexity, but have reduced immune risk. Each of them is culture-expanded, highlighting the importance of culture expansion for dose control and product reproducibility. Notably, no MSC-based therapy has been approved for OA in the USA, UK, or the European Union in publicly available regulatory databases at the time of manuscript acceptance.

Conversely, most of the cell therapy products for OA are being developed in Asia^139^ (Table 1). Although there is limited published evidence explicitly documenting a stronger cultural bias in Asian populations favoring joint preservation over knee arthroplasty, these patients often have higher functional demands for deep knee flexion post-TKA (for example, squatting and kneeling)^140^. Failure to meet these culturally influenced expectations may contribute to dissatisfaction, despite similar objective outcomes. Data available from Asian joint registries suggest that patients often undergo TKA at later stages of stiffness and degeneration^141^, implying a possible delay in opting for surgery, although causality is multifactorial. There is no robust published literature directly confirming that Asian patients generally prefer joint-preserving strategies over TKA, but there is suggestive evidence of delayed surgical uptake and ethnicity-linked differences in willingness to undergo TKA.

With most cell therapy products for OA being developed in Asia, and the sponsors having a geographically limited commercial infrastructure, the technologies may not make their way to Europe and the USA, unless a commercial partnership is formed. This is notable, as only ~10% of the current programs may have plans/ambitions for the European and American markets, as they appear to be focused on domestic population.

Besides cultural differences in the acceptance of TKA, other factors limiting the development of cell therapy products for OA in the USA and Europe include regulatory hurdles and limited funding. By contrast, for life-threatening diseases such as cancer, substantial investments and resources have led to the FDA approval of chimeric antigen receptor T cell therapy in hematologic malignancies^142^. Chimeric antigen receptor T cell therapy has demonstrated substantial improvements in overall survival among patients with poor prognosis^143^. However, in October 2025, three companies announced the discontinuation of their cell therapy programs, Galapagos, Novo Nordisk A/S, and Takeda Pharmaceutical Company, reflecting the modality risk of cell therapies, which are complex, expensive, often long-horizon, and manufacturing/logistics-intensive.

## Practical and operational challenges

### Manufacturing and standardization

A persistent challenge in advancing cell therapy products for OA is the complexity of manufacturing under good manufacturing practice (GMP) conditions. Differences in cell source (BM, adipose, and umbilical cord), isolation protocols, and expansion techniques contribute to notable variability in final cell products, raising concerns about reproducibility and comparability across studies^144,145^. Although considerable progress has been made in scaling up production, the field still lacks standardized methodologies that ensure consistent potency while minimizing batch-to-batch variation^146^. Establishing harmonized manufacturing processes will be critical for regulatory approval and global adoption.

### Supply chain challenges

The clinical implementation of cell therapy product is hampered by operational complexity. Manufacturing and distribution may require a cold chain infrastructure similar to mRNA vaccines or other ATMPs, including cell therapy products^13,147,148^. Cell therapy products are typically cryopreserved at –80 °C or lower, necessitating specialized handling protocols and trained personnel^149^. Clinicians and pharmacy/laboratory staff must be trained in handling cryopreserved products. Hospital pharmacy should provide liquid nitrogen (or equivalent storage) and trained technicians for thaw-and-deliver workflows. Clinics must manage cold chain receipt, thawing, administration, and documentation workflows. Decentralized, point-of-care, or cold chain-agnostic models are needed.

Most hospitals will have a cryobank/freezer for orthopedic use with tissue allografts, such as bone (for example, cortical, cancellous, and demineralized matrix) and soft tissue (for example, tendons, ligaments, menisci, and cartilage). As the cryobank is generally exclusive to the hospital (not clinic or ambulatory surgical center), cell therapy product is likely to be hospital-based initially.

### Logistical challenges

There is the logistical challenge of when clinicians may administer cell therapy product. Current stability parameters of cell therapy products would suggest a 48–72 h window after release from the sponsor/manufacturer for administration^150^. With next day shipping, this effectively limits the administration window to 24–48 h. The technique used is also critical, whether injection or surgical. For the latter, we would note that Medipost’s Cartistem requires arthrotomic implantation, whereas Celseed’s CLS2901C is implanted arthroscopically. The procedure or injection will need to confirm intra-articular placement for appropriate therapy and reimbursement.

### Superiority to orthobiologic comparators

From a clinical perspective, cell therapy products must show a meaningful advantage over orthobiologics such as PRP or BMAC. The desired characteristics include onset of action within 2–6 weeks, sustained benefit ≥6 months, structural improvements on imaging (for example, X-ray and MRI), potential to delay the time to surgery, and changes in key biochemical markers. Validated mechanistic biomarkers remain elusive, complicating both trial design and reimbursement.

## Regulatory and market considerations

### Regulatory frameworks

In the USA, culture-expanded allogeneic MSC therapies are regulated under Section 351 of the Public Health Service Act as biologics, requiring both an investigational new drug (IND) application and a biologics license application (BLA)^11,12^. Minimally manipulated autologous products may be eligible under Section 361 (refs. ^11,12^), although enforcement discretion has narrowed. The development of cell-based therapies tends toward minimally manipulated agents in the USA (six non-expanded agents out of eight and 0 expanded) (Tables 1 and 2), whereas Pacific-Asia leads the world in expanded cell therapy (25 expanded agents out of total 33 and 0 non-expanded) (Tables 1 and 2).

As of today, no MSC-based therapies for OA have been approved by European or American regulatory agencies; however, there are a few approved in Asia^139^. The FDA has approved one MSC-based product, Remestemcel-L from Mesoblast Corporation, for the treatment of steroid-refractory acute graft-versus-host disease (SR-aGVHD) utilizing allogeneic culture-expanded BM-MSCs^151,152^.

Regulatory agencies such as the EMA and the FDA identify pain and physical function as primary clinical outcomes for the treatment of OA^14,153^. By contrast, cancer-focused clinical trials involving cell therapies prioritize survival or progression-free survival as the primary end point, resulting in less emphasis and limited understanding of OA-related cell therapy trial outcomes^154^. For OA, this can be thought as survival of knee joint, without needing TKA.

### Regulatory hurdles and potency assays

From a regulatory perspective, the absence of validated potency assays remains a major obstacle. Current release criteria often focus on cell safety, viability, and phenotypic markers, which do not necessarily capture the paracrine or immunomodulatory functions thought to drive therapeutic benefit^13,155–159^. Regulatory authorities have emphasized the need for functional assays that reflect MoA, but consensus standards have yet to emerge. This gap complicates trial design, hinders comparability between studies, and introduces uncertainty into regulatory pathways for both autologous and allogeneic products.

The long-term potential for cell therapy products, both in terms of safety and efficacy, raises a couple of unique clinical and regulatory considerations. It will be difficult to randomize patients to a placebo and observe for 2–5 years without loss to follow-up, which increases the probability of an active control arm, while excluding the effect of concomitant therapies within this period. Regulatory expectations for clinical trials generally favor low attrition to preserve statistical power and validity. Although no specific numeric threshold is mandated, empirical reviews of RCTs commonly observe acceptable dropout rates in the ~10–20% range (for example, ~15%) to avoid substantial risk of bias^160–164^. In OA studies with longer follow-up durations and subjective patient-reported end points, dropouts can rise more steeply, which may limit feasible follow-up to a couple of years unless proactive retention strategies are used^161,164^. Sponsors should therefore engage with regulatory authorities early on trial design and plan for attrition risk, especially in smaller trials.

Unlike gene-modified therapies, MSCs offer non-integrating, transient action, reducing some long-term risks. However, regulatory agencies still require post-marketing surveillance. Sponsors may seek guidance from regulatory precedents in non-OA cell therapies, such as SR-aGVHD^151,152,165^, critical limb ischemia^166^, and fistula^167^.

Another unresolved regulatory issue is the rise of cell-free secretome and EV-based therapies that are yet to receive global and regulatory guidelines^168,169^. The only exception to this is the recent clinical guidance prepared together by the Japanese Society for Regenerative Medicine (JSRM) and the Japanese Society for Extracellular Vesicles (JSEV)^168,170^.

### Pricing and reimbursement speculations

If cell therapy products can reliably achieve ≥12 months of clinical effect and delay the need of TKA, there is flexibility around pricing. Partially due to the cost of manufacturing, storage, and distribution, pricing may fall between high-end orthobiologics (~$1,000)^171^^,^^172^ and surgical interventions such as TKA (~$20,000 in USA and ~10,000€ in European Union)^173,174^. A price above TKA may be acceptable for patients with early OA aged 40–55, who are not TKA candidates. Payers will require evidence of durable benefit and economic value across all OA types and ages. We would note the success of Vericel’s matrix-induced autologous chondrocyte implantation (MACI) in pricing above TKA in patients with chondral defect, where the therapy is typically used in patients younger than 55 years of age^175,176^.

In today’s global marketplace, tariffs represent an additional risk factor, as majority of cell therapy products are manufactured in Asia and Canada. Simply crossing international borders could increase drug prices.

### Logistics, scalability, and cost-effectiveness

Equally important are the logistical and economic considerations surrounding deployment of these therapies. Autologous approaches for expanded cells require individualized harvesting and processing, which limit scalability, whereas allogeneic strategies face challenges in storage, distribution, and immunological compatibility. Even if these issues are overcome, the high costs of cell procurement, processing, and administration raise questions about long-term cost-effectiveness, particularly when compared with lower-cost biologic injections such as HA or PRP^147,177^. Without robust health economic evidence and payer engagement, reimbursement is likely to remain limited to a subset of patients. Ultimately, successful adoption will require streamlined supply chains, harmonized reimbursement frameworks, and rigorous demonstration of both clinical efficacy and economic value^178^.

### Patient selection and access

Patient selection will be equally important for cell therapy products. The ideal candidate is likely to be younger (<55), lower grade of disease burden (KL 2–3), physically active, and not yet TKA-eligible (<55 years). Younger patients are also likely to have regenerative capacity preserved. This profile presents its own set of practical challenges. It may be challenging to identify and capture early progressing patients.

Unlike MACI, the ability to redose, similar to orthobiologics, is an open question for cell therapy products. In gene therapies, in which redosing owing to circulating antibodies against viral vectors may not be feasible^179^, cell therapy products hold the possibility for redosing^6^. Redosing is unlikely to be broadly supported by payors unless compelling evidence demonstrates sustained or additive clinical value. Overall, patient identification (those most likely to benefit), long-term tracking, and redosing protocols are future items to address^6^.

## Future directions and scientific gaps

The future clinical utility of cell therapy products for knee OA will be determined by the intersection of duration of effect and cost, rather than by mechanistic elegance alone. To justify their added complexity and high manufacturing costs, cell therapy products must demonstrate sustained clinical benefit of at least 12 months and ideally longer. Anything substantially shorter risks positioning these therapies as niche interventions, given the availability of simpler and less expensive options such as CSs, HA, PRP, and minimally manipulated autologous preparations.

Equally central to the value proposition is the potential for disease modification, operationally defined as a meaningful delay in (i) surgical eligibility (surgery-free progression) for TKA and (ii) time to TKA (joint survivorship) among patients progressing on the osteoarthritis journey. Future application would also benefit from identifying eligible patients earlier in the disease course, when the curve may be inflected, versus late-stage OA, when the curve may only be slowed at best.

These expectations have direct implications for trial design. Future pivotal studies will need to be larger and longer, with observation periods of at least 2–5 years to capture time to surgical eligibility (surgery-free progression) and progression to TKA (joint survivorship), alongside conventional pain (NRS/VAS) and function (WOMAC) end points. If a cell therapy product can show a robust and durable delay in surgical eligibility and/or TKA over such time frames, it may not require comparative trials against other injectables, because no existing intra-articular therapy has yet demonstrated this level of disease modification. By contrast, failure to demonstrate durable benefit or delay in surgery may relegate cell therapy products to “overengineered” solutions.

From a scientific perspective, improving durability may be a function of strategies that protect cells from the hostile OA joint environment and enhance local persistence. Promising avenues include biomaterial scaffolds, hydrogel-based or microcarrier formulations, and implant–cell combinations designed to improve localization, shield cells from mechanical and inflammatory stress, and prolong paracrine activity^180^. However, clinical evidence that such platforms meaningfully extend functional activity beyond that of cells alone remains limited, and carefully controlled studies will be required to validate these hypotheses. Parallel efforts in cell-free approaches (for example, EVs) may offer alternative routes to durable immunomodulatory and trophic effects with reduced logistical burden^181^.

Advances in cell engineering, including gene-modified cell therapies with the rise of technologies such as CRISPR–Cas systems^182–184^, iPSCs, and other manipulated cell-based platforms, are expanding the therapeutic landscape with powerful tools that can be customized for specific disease mechanisms^185,186^. Possibility of modifying cell-based platforms enables the researchers to overcome critical challenges, for example, in cell survival through arthritis-specific synthetic receptor systems^187^ and cell-free systems expressing modified EVs^188^. Although secretome and EV-based therapies promise more affordable and potentially safer solutions to cell therapy products, they face even more challenges in manufacturing consistency, potency assay design, and unclear regulatory and global frameworks^168,169^.

A major gap is the lack of standardized potency assays and release criteria^65,145,189^. Currently, there is no universally accepted measure of MSC “fitness” or biological activity. Regulatory agencies will require harmonized assays that capture the key mechanisms of action (for example, number of cells per dose, number of viable cells, immunogenicity related to number of cells, and secretome activity) to ensure reproducibility and comparability across trials. Addressing these gaps will be pivotal to advancing cell therapy products from experimental interventions to widely adopted, evidence-based treatments for OA.

Finally, the field is on the cusp of several pivotal clinical readouts that will likely shape future development priorities and investment. Late-stage programs such as gene-modified cell therapy (for example, TG-C) with ongoing phase III trials and anticipated readouts in the second half of 2026 will provide critical information on both durability and potential disease-modifying effects. The outcome of these and other large, long-term studies will help determine whether cell therapy products can substantively alter the OA treatment algorithm and inflect the OA curve, or whether they will be confined to a niche role in highly selected patient subsets in which simpler, lower-cost treatments are insufficient.

In summary, future progress will depend less on incremental mechanistic refinements and more on definitive, long-horizon clinical evidence that cell therapy products deliver durable symptom relief and delay surgery in a cost-justifiable manner. Without such data, the practical and economic hurdles may outweigh the theoretical advantages of these sophisticated modalities.

## Conclusions

Cell therapy products represent an intriguing but still unproven therapeutic frontier for knee OA, particularly for patients with KL grade 2–3 disease who lack durable non-surgical options. Although early clinical data suggest meaningful analgesic and functional improvements, the pathway to routine clinical adoption remains constrained by manufacturing complexity, regulatory uncertainty, operational burden, and payer skepticism. Ultimately, success will depend on whether these therapies can deliver reproducible, durable, and clinically meaningful benefit in the populations most likely to respond.

A central component of the value proposition for cell therapy products is duration of activity. To be competitive and justifiable relative to simpler orthobiologic comparators, such as autologous, minimally manipulated preparations, manipulated, cell-based interventions must demonstrate sustained clinical benefit measured in months and ideally years, rather than weeks. Equally important is the potential for disease modification: the ability to attenuate or slow structural progression and meaningfully delay surgical eligibility (surgery-free progression) and time to surgery (joint survivorship). Without evidence for durability and disease modification, cell therapy risks offering only incremental symptomatic benefit at substantially greater complexity and cost.

Biologically, cell therapy products possess compelling immunomodulatory and trophic mechanisms, but the hostile OA joint environment limits cell persistence and may curtail long-term effects. Next-generation strategies such as protective biomaterial carriers, scaffold-based delivery, or implant–cell combination systems seek to enhance localization, durability, and mechanistic potency. However, clinical evidence proving that such platforms meaningfully extend functional persistence remains limited, and a clear translational path is still emerging. Advances in gene-modified cell therapy, engineered secretomes, and cell-free EV products may further expand the therapeutic toolkit, but these innovations bring additional regulatory and operational complexity.

Finally, the field must remain cognizant of the possibility that cell therapy products and gene therapies, despite their sophistication, may represent an overengineered solution for a mechanically and biologically heterogeneous disease such as OA. In some scenarios, these approaches may violate Occam’s razor (that the simplest effective intervention is preferable), especially if they fail to outperform less complex, lower-cost alternatives, which so far do not demonstrate durable or disease-modifying benefits. However, this perspective must be balanced by the recognition that OA is a complex and multimodal disease, and this heterogeneity is likely why many previous, single-mechanism interventions have failed. It is this challenge that multitarget approaches such as cell-based and gene therapies are designed to address. Demonstrating durable, disease-modifying benefit is therefore not only a clinical necessity but also the cornerstone justification for adopting cell therapy products in OA.

## Supplementary information

Disclosure form Nancy Duarte-Delgado

Disclosure form Tobias Winkler

Disclosure form Jean-Yves Register

Disclosure form Ron Ellis

Disclosure form Nadia Benkirane-Jessel

Disclosure form Rana Smaida

Disclosure form Valtteri Peitso

Disclosure form Karman Ng

Disclosure form Ali Mobasheri

Disclosure form Simone Ponta

Disclosure form Goncalo Barreto
