# Supplementary material for: Next-generation therapies for osteoarthritis: the evolving role of cell therapy products
Source: Exp Mol Med. 2026 Jun 10;58(6):1754–71. doi: 10.1038/s12276-026-01726-y (PMC13324274; doi:10.1038/s12276-026-01726-y)

**ICMJE DISCLOSURE FORM**

**Date: 28.11.2025**

**Your Name: Prof. Tobias Winkler**

**Manuscript Title** Next-Generation Therapies for Osteoarthritis: The Evolving Role of Cell-Based Treatments

**Manuscript number (if known):__________________________________________________________________**

**In the interest of transparency, we ask you to disclose all relationships/activities/interests listed below that are**

**related to the content of your manuscript. “Related” means any relation with for-profit or not-for-profit third**

**parties whose interests may be affected by the content of the manuscript. Disclosure represents a commitment**

**to transparency and does not necessarily indicate a bias. If you are in doubt about whether to list a relationship/activity/interest, it is preferable that you do so.**

**The following questions apply to the** **author’s relationships/activities/interests as they relate to the current**

**manuscript only.**

**The author’s relationships/activities/interests should be defined broadly. For example, if your manuscript pertains**

**to the epidemiology of hypertension, you should declare all relationships with manufacturers of antihypertensive medication, even if that medication is not mentioned in the manuscript.**

**In item #1 below, report all support for the work reported in this manuscript without time limit. For all other items,**

**the time frame for disclosure is the past 36 months.**

|  |  | **Name all entities with whom you have this relationship or indicate none (add rows as needed)** | | **Specifications/Comments**  **(e.g., if payments were made to you or to your institution)** |
| --- | --- | --- | --- | --- |
| **Time frame: Since the initial planning of the work** | | | | |
| 1 | All support for the present manuscript (e.g., funding, provision of study materials, medical writing, article processing charges, etc.)  **No time limit for this item.** | European Commission | | EU Horizon Europe Program, PROTO project, nr. 101095635, funded under HORIZON-HLTH-2022-STAYHLTH-02.01 |
|  |  |  | |  |
|  |  |  | |  |
|  |  |  | |  |
|  |  |  | |  |
|  |  |  | |  |
|  |  |  | |  |
|  |  |  | |  |
| **Time frame: past 36 months** | | | | |
| 2 | Grants or contracts from any entity (if not indicated in item #1 above). | European Commission | HIPGEN EU Horizon Programm Nr. 779293 | |
|  |  | BMBF Germany | CRC funding CRC 1444 | |
|  |  | Innovation Fund Denmark | Research Funding | |
| 3 | Royalties or licenses | __x__None |  | |
|  |  |  |  | |
|  |  |  |  | |
| 4 | Consulting fees | Liposphere |  | |
|  |  | Relive |  | |
|  |  | Enlivex |  | |
|  |  | Pluri |  | |
|  |  | Ossium |  | |
|  |  | Heraeus Medical |  | |
| 5 | Payment or honoraria for lectures, presentations, speakers bureaus, manuscript writing or educational events | __x__None |  | |
|  |  |  |  | |
|  |  |  |  | |
| 6 | Payment for expert testimony | __x__None |  | |
|  |  |  |  | |
|  |  |  |  | |
| 7 | Support for attending meetings and/or travel | _x___None |  | |
|  |  |  |  | |
|  |  |  |  | |
| 8 | Patents planned, issued or pending | __x__None |  | |
|  |  |  |  | |
|  |  |  |  | |
| 9 | Participation on a Data  Safety Monitoring Board or Advisory Board | _x___None |  | |
|  |  |  |  | |
|  |  |  |  | |
| 10 | Leadership or fiduciary role in other board, society, committee or advocacy group, paid or unpaid | Advanced Therapies in Orthopaedics Foundation | Executive Board | |
|  |  |  |  | |
|  |  |  |  | |
| 11 | Stock or stock options | Sunac, Myotec, Actimed, Tissue Connect Systems |  | |
|  |  |  |  | |
|  |  |  |  | |
| 12 | Receipt of equipment, materials, drugs, medical writing, gifts or other services | __x__None |  | |
|  |  |  |  | |
|  |  |  |  | |
| 13 | Other financial or non-financial interests | _x___None |  | |
|  |  |  |  | |
|  |  |  |  | |

**Please place an “X” next to the following statement to indicate your agreement:**

**__x_ I certify that I have answered every question and have not altered the wording of any of the questions on this**

**form.**


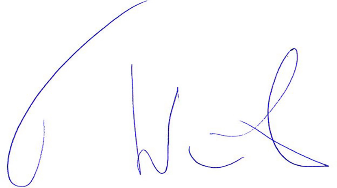

Supplement: Supplementary file 2 — Disclosure form Tobias Winkler [file 12276_2026_1726_MOESM2_ESM.docx]
