# Supplementary material for: Next-generation therapies for osteoarthritis: the evolving role of cell therapy products
Source: Exp Mol Med. 2026 Jun 10;58(6):1754–71. doi: 10.1038/s12276-026-01726-y (PMC13324274; doi:10.1038/s12276-026-01726-y)
Supplement: Supplementary file 9 — Disclosure form Ali Mobasheri [file 12276_2026_1726_MOESM9_ESM.docx]

| ICMJE DISCLOSURE FORM | |
| --- | --- |
| **Date:** | 11/28/2025 |
| **Your Name:** | Ali Mobasheri |
| **Manuscript Title:** | Next-Generation Therapies for Osteoarthritis: The Evolving Role of Cell-Based Treatments |
| **Manuscript Number (if known):** | Click or tap here to enter text. |
| In the interest of transparency, we ask you to disclose all relationships/activities/interests listed below that are related to the content of your manuscript. “Related” means any relation with for-profit or not-for-profit third parties whose interests may be affected by the content of the manuscript. Disclosure represents a commitment to transparency and does not necessarily indicate a bias. If you are in doubt about whether to list a relationship/activity/interest, it is preferable that you do so.  The author’s relationships/activities/interests should be defined broadly. For example, if your manuscript pertains to the epidemiology of hypertension, you should declare all relationships with manufacturers of antihypertensive medication, even if that medication is not mentioned in the manuscript.  In item #1 below, report all support for the work reported in this manuscript without time limit. For all other items, the time frame for disclosure is the past 36 months. | |

|  | | | **Name all entities with whom you have this relationship or indicate none (add rows as needed)** | **Specifications/Comments (e.g., if payments were made to you or to your institution)** |
| --- | --- | --- | --- | --- |
| **Time frame: Since the initial planning of the work** | | | | |
| **1** | All support for the present manuscript (e.g., funding, provision of study materials, medical writing, article processing charges, etc.)  **No time limit for this item.** | | \|  \| **None** \| \| --- \| --- \|  \| Academy of Finland (Research Council of Finland) \| Research Council of Finland/Suomen Akatemia Profi 6, grant numbers 336449 and 351568, awarded to Ali Mobasheri at the University of Oulu. \| \| --- \| --- \| \|  \|  \| \|  \| Click the tab key to add additional rows. \| | |
| **Time frame: past 36 months** | | | | |
| **2** | | Grants or contracts from any entity (if not indicated in item #1 above). | \|  \| **None** \| \| --- \| --- \|  \| European Commission  Academy of Finland (Research Council of Finland)  Research Council of Lithuania (Lietuvos Mokslo Taryba)  COST Association \|  \| \| --- \| --- \| \|  \|  \| \|  \|  \| | |
| **3** | | Royalties or licenses | \|  \| **None** \| \| --- \| --- \|  \|  \|  \| \| --- \| --- \| \|  \|  \| \|  \|  \| | |
| **4** | | Consulting fees | \|  \| **None** \| \| --- \| --- \|  \| ACI, Active Health GmbH, Ampio Pharmaceuticals, Apos Health, Aptissen SA, argenx, Banyan Biotechnology, BioSplice Therapeutics, Bruder Consulting and Venture Group, California Institute for Regenerative Medicine (CIRM), Chiron Organ-on-Chip Systems, Chondrometrics GmbH, Chondropeptix, Contura, Enlivex, Genascence, Grünenthal, GSK, HALEON, Hypera Pharma, IBSA, ICM, Image Analysis Group (IAG), Kangstem, Kolon Life Science, Kolon TissueGene, Laboratoires Expanscience, Mars, Waltham, McKinsey and Company, Nestlé Health Science, Pacira Biosciences, Pleryon, Pluri, Relevium Medical, Rottapharm Biotechnology, Sanofi, Sanofi CHC / Opella, Sporlastic GmbH, Sunac Therapeutics, Synartro AB, SynOA Therapeutics, and Viatris. \|  \| \| --- \| --- \| \|  \|  \| \|  \|  \| \|  \|  \| | |
| **5** | | Payment or honoraria for lectures, presentations, speakers bureaus, manuscript writing or educational events | \|  \| **None** \| \| --- \| --- \|  \| Janssen, Viatris, Grünenthal, Novartis, Orion Corporation \|  \| \| --- \| --- \| \|  \|  \| \|  \|  \| | |
| **6** | | Payment for expert testimony | \|  \| **None** \| \| --- \| --- \|  \|  \|  \| \| --- \| --- \| \|  \|  \| \|  \|  \| | |
| **7** | | Support for attending meetings and/or travel | \|  \| **None** \| \| --- \| --- \|  \| Janssen Cilag (Brazil), Viatris, Opella, Hypera \|  \| \| --- \| --- \| \|  \|  \| \|  \|  \| | |
| **8** | | Patents planned, issued or pending | \|  \| **None** \| \| --- \| --- \|  \|  \|  \| \| --- \| --- \| \|  \|  \| \|  \|  \| | |
| **9** | | Participation on a Data Safety Monitoring Board or Advisory Board | \|  \| **None** \| \| --- \| --- \|  \|  \|  \| \| --- \| --- \| \|  \|  \| \|  \|  \| | |
| **10** | | Leadership or fiduciary role in other board, society, committee or advocacy group, paid or unpaid | \|  \| **None** \| \| --- \| --- \|  \| Osteoarthritis Research Society International  European Society for Clinical and Economic Aspects of Osteoporosis, Osteoarthritis and Musculoskeletal Diseases (ESCEO) \| I have served as President of the Osteoarthritis Research Society International (OARSI) (May 2019 - May 2022) and a member of the OARSI Board of Directors from 2013-2022. I currently serve as an advisor for the World Health Organization Collaborating Center for Public Health Aspects of Musculoskeletal Health and Aging, Member-at-Large on the Steering Committee Osteoarthritis Action Alliance, and Member of the Scientific Advisory Board of the European Society for Clinical and Economic Aspects of Osteoporosis, Osteoarthritis and Musculoskeletal Diseases (ESCEO) \| \| --- \| --- \| \|  \|  \| \|  \|  \| | |
| **11** | | Stock or stock options | \|  \| **None** \| \| --- \| --- \|  \| Sunac Therapeutics \| Shares \| \| --- \| --- \| \|  \|  \| \|  \|  \| | |
| **12** | | Receipt of equipment, materials, drugs, medical writing, gifts or other services | \|  \| **None** \| \| --- \| --- \|  \| Chiron \| Organ-on-chip devices \| \| --- \| --- \| \|  \|  \| \|  \|  \| | |
| **13** | | Other financial or non-financial interests | \|  \| **None** \| \| --- \| --- \|  \| Ali Mobasheri currently consults for the following cell therapy companies: Kangstem, Pluri, Enlivex, Kolon TissueGene \|  \| \| --- \| --- \| \|  \|  \| \|  \|  \| | |
|  | |  |  | |
| **Please place an “X” next to the following statement to indicate your agreement:** | | | | |
|  | | I certify that I have answered every question and have not altered the wording of any of the questions on this form. | | |
